# Generating Actionable Data From Antimicrobial Resistance Surveillance: Insights From the Fleming Fund's International Experience

**DOI:** 10.1093/cid/ciag497

**Published:** 2026-10-19

**Authors:** Ankur Gupta-Wright, Suliat Yetunde Adeleke, Victoria Adetunji, Qadeer Ahsan, Ritu Amatya, Ben Amos, Brian Asare, Sorie I O Bangura, Cyril Buhler, Nicholas Feasey, N Claire Gordon, Heidi Hopkins, Freddy Eric Kitutu, Toby Leslie, Ibrahima Lo, Seynabou Lo, Ayodele Majekodunmi, Rebecca Maudling, Ntombi B Mudenda, Victor Moses Musyoki, Deborah Nanjebe, Tessa Oakley, Manish Pathak, Tamalee Roberts, Lamiaa Shehata, Tim Shorten, Nure Alam Siddiky, John Stelling, Muhammad Taimoor, May Soe Thwe, Shawn Ting, Didem Torumkuney, Paul Turner, Jacob Wildfire, Timothy David Worth

**Affiliations:** Department of Infectious Disease, Imperial College London, London, United Kingdom; Department of Infectious Diseases and Tropical Medicine, Heidelberg University Hospital, Heidelberg, Germany; Department of Infectious Diseases, Bristol NHS Foundation Trust, Bristol, United Kingdom; Itad Ltd, Brighton, United Kingdom; Federal Ministry of Livestock Development, Abuja, Nigeria; Department of Veterinary Public Health and Preventive Medicine, University of Ibadan, Ibadan, Nigeria; DAI Global LLC, Bethesda, Maryland, USA; FHI 360, Kathmandu, Nepal; Independent consultant; Pharmacy Directorate, Ministry of Health, Accra, Ghana; Ministry of Health and Sanitation, Freetown, Sierra Leone; ORDiagnostics, France; The School of Medicine, University of St Andrews, St Andrews, United Kingdom; Department of Clinical Sciences, Liverpool School of Tropical Medicine, Liverpool, United Kingdom; UK Health Security Agency, Porton Down, Salisbury, United Kingdom; Fleming Fund , International Development Sector, Mott MacDonald, London, United Kingdom; Department of Pharmacy, School of Health Sciences, Makerere University College of Health Sciences, Kampala, Uganda; Department of Women’s and Children’s Health, International Child Health and Migration, Uppsala University, Uppsala, Sweden; Fleming Fund , International Development Sector, Mott MacDonald, London, United Kingdom; Food and Agriculture Organization of the United Nations, Dakar, Senegal; UFR des Sciences de la Santé, Université Gaston Berger de Saint-Louis, Saint-Louis, Senegal; Ajisefini Consulting, London, United Kingdom; Itad Ltd, Brighton, United Kingdom; Independent consultant; Department of Clinical Studies, School of Veterinary Medicine, University of Zambia, Lusaka, Zambia; Burnet Institute, Melbourne, Victoria, Australia; Fleming Fund , International Development Sector, Mott MacDonald, London, United Kingdom; Global and Tropical Health Division, Menzies School of Health Research, Charles Darwin University, Darwin, Northern Territory, Australia; Fleming Fund , International Development Sector, Mott MacDonald, London, United Kingdom; Lao-Oxford-Mahosot Hospital-Wellcome Trust Research Unit, Mahosot Hospital, Vientiane, Lao People's Democratic Republic; Centre for Tropical Medicine and Global Health, Nuffield Department of Medicine, University of Oxford, Oxford, United Kingdom; Itad Ltd, Brighton, United Kingdom; Itad Ltd, Brighton, United Kingdom; Independent consultant; DAI Global LLC, Bethesda, Maryland, USA; Department of Medicine, Division of Infectious Diseases, Brigham and Women’s Hospital, Boston, Massachusetts, USA; DAI Global LLC, Bethesda, Maryland, USA; Fondation Mérieux, Vientiane, Lao People's Democratic Republic; Global and Tropical Health Division, Menzies School of Health Research, Charles Darwin University, Darwin, Northern Territory, Australia; GSK, London, United Kingdom; Centre for Tropical Medicine and Global Health, Nuffield Department of Medicine, University of Oxford, Oxford, United Kingdom; Cambodia Oxford Medical Research Unit, Angkor Hospital for Children, Siem Reap, Cambodia; Fleming Fund , International Development Sector, Mott MacDonald, London, United Kingdom; Fleming Fund , International Development Sector, Mott MacDonald, London, United Kingdom

**Keywords:** antimicrobial resistance, surveillance, laboratory capacity, antimicrobial susceptibility testing, low- and middle-income countries

## Abstract

Antimicrobial resistance (AMR) is a major global health threat, with the greatest burden falling on low- and middle-income countries (LMICs) also challenged with limited surveillance capacity. The UK Government's Fleming Fund was established in 2015 to strengthen AMR surveillance across Africa and Asia through a One Health approach. This paper draws on program experience, country implementation evidence, and published literature to describe how the Fleming Fund operationalized AMR surveillance, assess its contribution to the quantity and quality of surveillance data, identify key implementation challenges, and consider implications for future global surveillance strategies. The Fleming Fund combined investments in laboratory infrastructure, workforce development, quality assurance, data systems, governance, and data use. Across supported countries, these investments enabled substantial growth in routine bacteriology and antimicrobial susceptibility testing, strengthened reference and surveillance laboratory capacity, increased participation in external quality assessment, and expanded reporting to the World Health Organization's Global Antimicrobial Resistance and Use Surveillance System. The program also supported complementary surveillance approaches, including antimicrobial use and consumption data and, in some settings, burden-of-disease and genomic surveillance. Progress, however, has been uneven and remains vulnerable to fragile infrastructure, procurement and supply chain bottlenecks, workforce turnover, fragmented data systems, weak clinical–laboratory interfaces, limited representativeness, and uncertain long-term financing. The Fleming Fund experience shows that strengthening AMR surveillance in LMICs is feasible, but requires sustained, system-wide investment. Future strategies should prioritize integration with clinical care, stronger digital and analytical capacity, broader diagnostic access, and domestic financing to ensure that high-quality AMR data are generated, shared, and translated into action.

Antimicrobial resistance (AMR) is a major global health threat, causing excess morbidity and mortality by reducing the effectiveness of antimicrobial therapy, as well as negative impacts on food security and economies, with its burden falling disproportionately on low- and middle-income countries (LMICs) [1–4]. This burden is forecast to increase substantially, with an estimated 1.9 million (95% CI 1.6–2.3) attributable and 8.2 million (95% CI 6.9–9.7) associated deaths by 2050, although modeled estimates are largely driven by data from high-income countries [5].

The Fleming Fund was established in 2015 by the UK Government's Department of Health and Social Care to tackle the AMR crisis in LMICs in Africa and Asia through a One Health approach, comprising coordinated, multisectoral, and multidisciplinary action across human, animal, and environmental health systems [6]. A central objective of the World Health Organization (WHO) 2015 Global Action Plan on AMR was to strengthen knowledge and evidence through surveillance, which was a core deliverable for many AMR national action plans [7–9]. The Global Antimicrobial Resistance and Use Surveillance System (GLASS) was launched by WHO in October 2015 as the first global effort to standardize AMR surveillance approaches and enable cross-country comparability [10].

In many settings, the key bottleneck to generating actionable AMR surveillance data was largely driven by the need for quality-assured laboratory systems to identify AMR, mechanisms for data harmonization and analysis, and systems capable of producing and using reliable AMR surveillance data at scale. Data are essential across all AMR actions, including development of treatment guidelines, infection prevention and control, and informing antimicrobial stewardship (AMS) strategies. At the policy level, robust surveillance data should underpin national action plans and prioritization of limited resources and enable governments and partners to target investments where AMR burden and health system gaps are greatest, ensuring accountability and measurable impact.

An early output from the Fleming Fund investment was the London School of Hygiene & Tropical Medicine AMR surveillance roadmap, which articulated AMR as requiring coordinated investments across laboratories, data, governance, and use [11]. Delivered through country, regional, and fellowship grants, as well as support to multilateral organizations, the Fleming Fund investment across 2 phases and 10 years totaled nearly £500 m and focused on strengthening routine bacteriology and antimicrobial susceptibility testing (AST), data capture and analysis, and embedding surveillance within national AMR action plans across human and animal health sectors (Figure 1) [6, 12]. Phase 2 built on foundations, with a stronger emphasis on sustainability, data use for policy and practice, antimicrobial use and consumption data, and integration of individual-level and aggregate surveillance data within broader health system functions [6].

**Figure 1.**
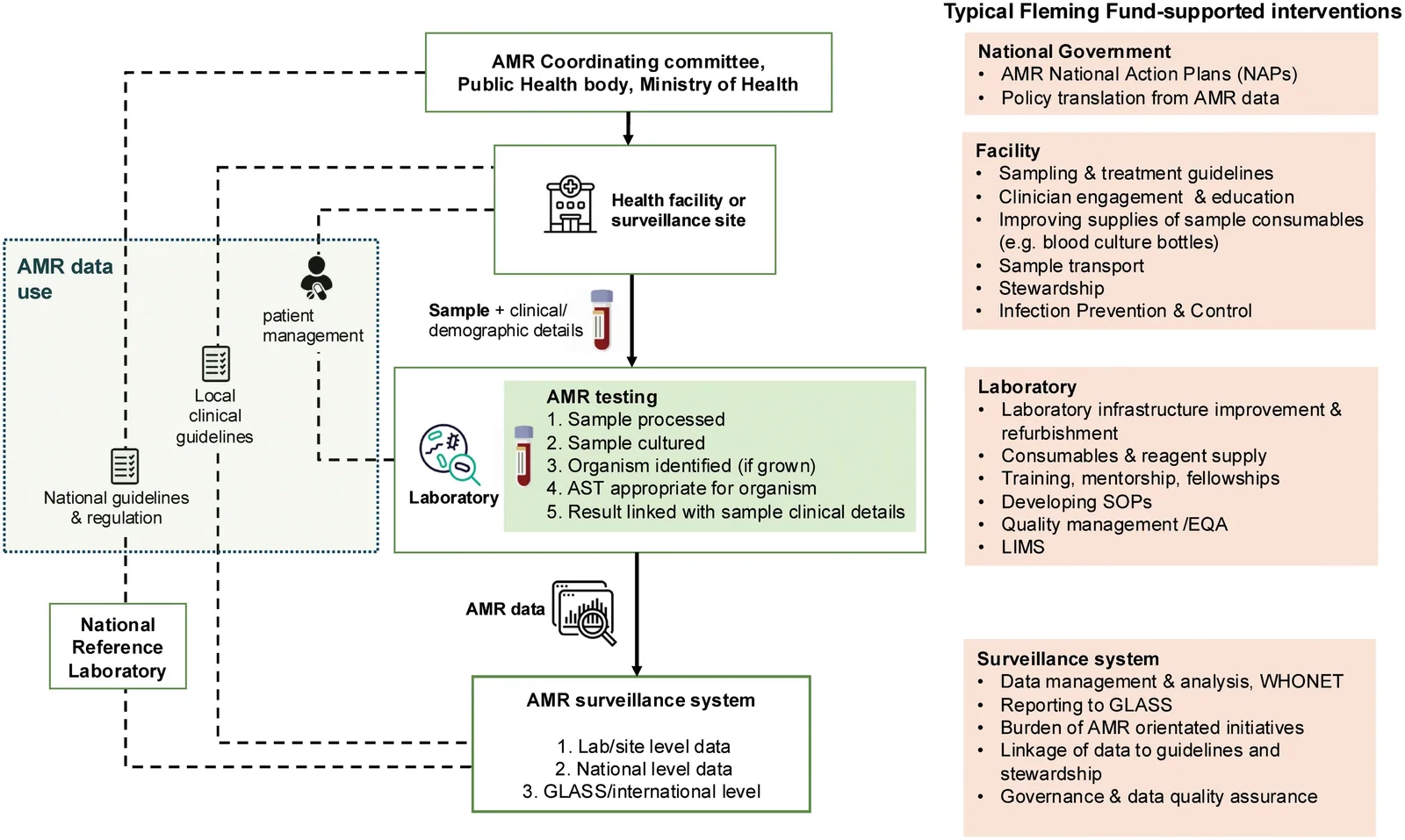
Overview of AMR data generation at surveillance site/health facility and higher levels and Fleming Fund interventions to support data generation. Abbreviations: AMR, antimicrobial resistance; AST, antimicrobial susceptibility testing; EQA, external quality assessment; GLASS, Global Antimicrobial Resistance and Use Surveillance System; LIMS, laboratory information management systems; NAP, national action plan; SOP, standard operating procedure.

This paper is part of a journal series synthesizing the evidence and lessons from the Fleming Fund to contribute to wider learning on how complex global health programs translate technical capacity into institutional and policy change. The aims are to (1) describe how the Fleming Fund operationalized AMR surveillance, (2) describe changes in the quantity and quality of AMR data, (3) describe how the Fleming Fund has addressed challenges faced in implementing AMR data surveillance, and (4) consider the implications of this experience for strengthening global AMR surveillance. This paper aims to draw directly from Fleming Fund experiences and lessons, be grounded in practitioner perspectives, provide actionable guidance, and examine plausible contribution pathways and implementation lessons. Although the Fleming Fund was a One Health program and included capacity building in the animal and environment sectors [13], the majority of the investment was within the human health sector, which is the main focus of this paper.

## METHODS

This paper forms part of a practice-informed series synthesizing learning from Fleming Fund-supported AMR surveillance strengthening activities. It draws on program documentation, evaluation evidence, relevant literature, and structured engagement with a broad range of contributors from multiple disciplines and perspectives, including implementing countries and the wider AMR community. The series does not constitute an independent evaluation or seek to establish causal attribution. Findings should therefore be interpreted in light of limitations, including program-linked authorship, contributor perspectives, and purposive source selection.

The Fleming Fund delivered context-specific packages of interventions across countries, combining laboratory strengthening with workforce development, governance, and data use. Examples from Timor-Leste, Ghana, and Nepal illustrate how these investments translated into AMR surveillance system gains.

## RESULTS

### How Did the Fleming Fund Operationalize Antimicrobial Resistance Surveillance Data Generation?

The Fleming Fund was designed to support AMR data generation through an approach that linked laboratory capacity, workforce development, surveillance practice, and data use (Figure 1; Box 1). At the country level, investments focused first on strengthening laboratory infrastructure to enable testing of WHO priority pathogen–drug combinations (Table 1) [8]. This included renovation of microbiology laboratories; provision and installation of laboratory equipment, including incubators, biosafety equipment, automated blood culture, and identification and AST platforms; provision of reliable supplies of reagents and consumables; and development of standard protocols (Table 1; Box 2). Human resource strengthening accompanied infrastructure support through training of clinicians, laboratory scientists, microbiologists, epidemiologists, and data managers, including a “Fleming Fellowship” scheme, which supported individualized high-level training for fellows within national reference laboratories, surveillance sites, and policy institutions to build sustainable technical and leadership capacity. However, the support varied significantly between participating countries.

Box 1. **Examples of data generated by the Fleming Fund for antimicrobial resistance surveillance, including antimicrobial use and antimicrobial consumption**

The Fleming Fund supported the generation of complementary data streams that underpin antimicrobial resistance (AMR) surveillance.

**Antimicrobial resistance:** AMR data are derived from routine microbiological testing at surveillance site and national reference laboratories, primarily through culture of samples from patients, animals, and the environment, followed by antimicrobial susceptibility testing (AST) of isolates. Active surveillance was also used in animal health, eg sampling from farms and abattoirs. Strengthening of laboratory systems has enabled increased processing of patient and animal samples and improved data quality through standardized methods and external quality assurance.

**Antimicrobial use (AMU):** AMU data describe prescribing practices and are typically generated through cross-sectional point prevalence surveys (PPS) in hospitals and other healthcare settings, capturing indications, drug choice, and appropriateness of use through standardized methodologies. In the animal health sector, AMU data may be collected through farm-level surveys. These data provide critical insights into prescribing behavior and are widely used to target antimicrobial stewardship interventions.

**Antimicrobial consumption (AMC):** AMC data quantify antimicrobial volumes at the population level, most commonly derived from pharmaceutical import, sales, or distribution data. While less granular than AMU data, AMC provides a system-level view of antimicrobial exposure and is useful for benchmarking, tracking trends over time, and monitoring the impact of national stewardship policies.

**Burden of disease data:** At the facility level in some sites, burden of disease data are generated through prospective clinical surveillance platforms or protocols (eg A Clinically Oriented Antimicrobial Resistance Surveillance Network [ACORN] and BALANCE). These systems link patient-level clinical data (eg syndrome, comorbidities, treatment, and outcomes) with microbiological results to estimate the incidence and outcomes of drug-resistant infections in defined populations. Unlike routine laboratory surveillance, these approaches enable attribution of patient-relevant outcomes, such as mortality, length of stay, and treatment failure to resistant pathogens. Such data are particularly valuable for understanding the impact of AMR, but are distinct from routine surveillance. However, the integration of clinical data into routine surveillance will increase the potential for policy impact.

Box 2. **Examples of Fleming Fund implementation and impact in selected countries**

In **Timor-Leste**, 2 country grants supported **8 laboratories** (7 human health and 1 animal health), including the National Health Laboratory as a reference center. Interventions included laboratory renovation, provision of equipment, implementation of laboratory information management systems (LIMS), and standard operating procedures across all sites. These investments enabled routine bacterial identification and AST and supported the country's **first reporting to GLASS (2021)** and **first reporting** of **antimicrobial consumption data to WOAH (2020)**. Capacity building was reinforced through **12 fellowships**, with trained microbiologists and epidemiologists embedded in laboratories, alongside policy fellows supporting governance. Additional activities included establishment of AMR data systems, point prevalence surveys, and multisectoral coordination through a national committee, contributing to the development of prescribing guidelines and AMR policy processes.

In **Ghana**, the Fleming Fund supported **11 laboratories** (8 human and 3 animal health), alongside investments in governance and data systems. Laboratory strengthening included equipment provision (eg blood culture systems, matrix-assisted laser desorption/ionization–time of flight [MALDI-TOF], automated AST platforms), quality management systems, and participation in external quality assurance schemes. Beyond laboratories, the program supported development of a **national AMR surveillance strategy**, point prevalence surveys in hospitals, and establishment of antimicrobial consumption databases. **Twelve fellowships** and multiple regional grants contributed to workforce development and data analysis capacity. These combined efforts enabled Ghana to progress from no operational AMR surveillance system to a **moderate, multisectoral system with GLASS reporting and national data use structures**.

In **Nepal**, investments of over £4 million supported **27 laboratories** (17 human, 7 animal, and 2 food safety, and 1 environmental sector laboratory), alongside extensive system-level strengthening. Interventions included laboratory renovation, provision of equipment and consumables, and implementation of quality assurance systems across sites. Surveillance capacity was expanded through development of national protocols; initiation of active and passive surveillance in humans, animals, and food; and integration of data systems (including WHONET and DHIS2). **Twelve fellowships** supported technical, analytical, and policy capacity. Additional activities included repeated point prevalence surveys, nationwide antimicrobial use surveys in poultry, and development of digital systems for AMU/AMC data. These investments contributed to Nepal's transition to a **more advanced surveillance system**, with a demonstrated influence on treatment guidelines and essential medicines policy.

**Table 1.** Main Components of the AMR Surveillance System Strengthened by the Fleming Fund, With Illustrative Interventions, Rationale, and Challenges

AMR Surveillance System Component	Fleming Fund Interventions	Why This Mattered	Persistent Challenges
Laboratory strengthening: infrastructure and diagnostic microbiology	Renovation of laboratories; improvements to electricity, water, and biosafety; provision of microbiology equipment (eg incubators, blood culture systems, and identification and AST platforms); technical support to national reference laboratories and surveillance sites; provision of consumables and reagents with centralized or pooled procurement; biorepository development	Enabled routine bacteriology and antimicrobial susceptibility testing in settings where this was previously not available; increased testing capacity and number of functional sites; underpinned generation of AMR surveillance data and clinical microbiology services	Reliance on stable utilities and supply chains; high recurrent costs (reagents, maintenance especially advanced equipment)
Quality assurance and standardization	Strengthening of quality management systems through development and implementation of SOPs and laboratory protocols; participation in EQA schemes; support to reference laboratory oversight; alignment with GLASS and national surveillance standards; regional quality assurance initiatives	Improved reliability, comparability, and credibility of microbiology results; enabled aggregation of data across sites and countries	Variable implementation of quality systems; limited accreditation; dependence on sustained supervision and resources
Training, mentorship, and workforce development	In-service training for clinicians, laboratory staff, and other personnel involved in surveillance; mentorship programs; fellowships in microbiology, epidemiology, governance, and policy; regional training initiatives; online training platforms; development of communities of practice	Strengthened technical capacity and workforce competence; improved testing quality and data interpretation; built a cadre of professionals linking laboratory work to surveillance and policy	High staff turnover; institutionalization of training; need for repeated capacity building; career pathways, and retention mechanisms
Sample pathways and clinical interface	Strengthening specimen referral pathways; support to surveillance site networks; clinician–laboratory engagement; feedback of results; integration with infection prevention and control and antimicrobial stewardship activities	Increased appropriate specimen collection and improved clinical relevance of data; strengthened links between laboratory outputs and patient care; supported facility-level use of surveillance data	Variable demand for microbiology services; clinician trust or awareness; turnaround time; affordability barriers; utilization of laboratory capacity
Data capture and management	Implementation of WHONET for microbiology data analysis; implementation of LIMS; national data aggregation platforms; dashboards and reporting tools; retrospective data mapping (eg MAAP); support for reporting to GLASS; support to AMU/AMC data collection, including point prevalence surveys	Enabled transformation of site-level laboratory data into national surveillance datasets; improved reporting to international systems; supported routine analysis and dissemination of AMR data	Fragmented data systems; incomplete metadata and missing variables; integrating AMR, AMU, and AMC data; limited automation in some settings
Data analysis and interpretation	Production of national surveillance reports; development of facility-level antibiograms; training in data analysis; dashboards and visualization tools; early work on burden of disease and economic analysis	Facilitated interpretation of AMR trends; supported clinical decision-making and stewardship; provided evidence for policy and advocacy; increased visibility of AMR burden	Analytical capacity; greater focus on AMR than AMU/AMC; transition from descriptive to actionable analysis; routine use of outputs
Governance, coordination, and policy translation	Support to AMR coordinating committees and technical working groups; development of national action plans; support for convening and stakeholder coordination; strengthening links between surveillance and policy processes; engagement with international organizations (WHO, FAO, WOAH); fellowships focused on policy and governance	Created institutional structures to oversee surveillance and data use; improved coordination across sectors; enabled data sharing and discussion at the national level; contributed to the enabling environment for AMR action	Fragmented governance structures in some countries; limited translation of data into policy and practice; dependence on political prioritization; coordination challenges across multiple actors and sectors
Genomics and advanced reference functions	Support for whole-genome sequencing capacity through regional grants; provision of sequencing platforms; training in bioinformatics; strengthening of reference laboratory functions; integration with surveillance systems in selected contexts	Enabled deeper understanding of AMR mechanisms and transmission; supported outbreak investigation and advanced surveillance; strengthened regional technical capacity	High technical and financial requirements; limited bioinformatics capacity; sustainability challenges

Abbreviations: AMC, antimicrobial consumption; AMR, antimicrobial resistance; AMU, antimicrobial use; AST, antimicrobial susceptibility testing; EQA, external quality assessment; FF, Fleming Fund; FAO, Food and Agriculture Organization; GLASS, Global Antimicrobial Resistance and Use Surveillance System; LIMS, laboratory information management systems; NAP, national action plan.

To ensure high-quality data are generated for decision-making at the facility level and higher levels, the Fleming Fund supported implementation of laboratory quality management systems, including external quality assessment (EQA), standard operating procedure (SOP) development, and training and progression toward laboratory accreditation [14–17]. Country-level investments were reinforced by regional grants supporting EQA in Africa and Asia, strategic alignment grants, and direct grants to multilateral partners to strengthen global surveillance standards and platforms, thereby linking national data production to regional and global AMR structures [6].

Surveillance was also operationalized through diagnostic stewardship (ie efforts to improve sampling and the appropriate use of microbiological diagnostics), for example supporting guidelines for patient selection criteria, appropriate specimen collection with adequate clinical and demographic information (aligned with GLASS), active microbiological sampling of animals and the environment in selected contexts, and closer integration of laboratories with clinicians, stewardship committees, and AMR coordination and governance mechanisms. Data management systems were strengthened via introduction or upgrading of electronic data management and analysis systems (eg laboratory information management systems [LIMS] and tools such as WHONET), and structured reporting at the laboratory or facility and national levels, and to international platforms, such as GLASS [18, 19].

In recognition that isolate-based sentinel site AMR surveillance programs provide an incomplete picture of AMR burden, as data reflect testing practices and laboratory capacity as much as true disease incidence, the Fleming Fund also supported burden-of-disease data generation and modeling. The Global Research on Antimicrobial Resistance (GRAM) demonstrated that current modeling estimates rely heavily on high-income country data due to limited availability of linked clinical–microbiological datasets from LMICs, with major uncertainties driven by incomplete data coverage and surveillance biases. In response, Phase 2 of the Fleming Fund emphasized engagement between the laboratories and healthcare professionals taking diagnostic samples, and seeking bacteriology results, and supporting standardized tools, such as A Clinically Oriented Antimicrobial Resistance Surveillance Network, Burden of Antibiotic Resistance in Neonates from Developing Societies, BALANCE, and Technique de Surveillance Actualisée de la Résistance aux Antimicrobiens through regional technical assistance programs [20–25].

### Has the Fleming Fund Supported the Generation of Antimicrobial Resistance Surveillance Data?

Prior to the Fleming Fund, just over half of the Fleming Fund countries had a formal AMR surveillance system in place for human health, but few Fleming Fund-supported laboratories were generating and reporting high-quality AMR data [26–28]. There were also other programs supporting AMR surveillance in LMICs and reporting data, including WHO regional AMR surveillance networks (e.g. WHO Pan American Health Organization's Latin American and Caribbean Network for Antimicrobial Resistance Surveillance, ReLAVRA) and research-led programs (eg Wellcome Trust Major International Programs and INSPEAR) [29–34]. There were also several well established hospital-based sentinel surveillance sites in LMICs (eg India's ICMR-AMRSN, Vietnam's VINARES, China's CARSS, and South Africa's GERMS-SA) [35–38].

Since implementation, there has been a substantial increase in the overall amount of AMR data generated by 335 Fleming Fund-supported sites across all regions (Figures 2–4). In the human health sector, 6.9 million samples have been processed in 25 countries, with the largest increases seen in Phase 2 since 2022, particularly for blood cultures (2.6 million samples processed; Figure 2). Fleming Fund-supported countries reporting to GLASS increased from 8 in 2018 to 18 in 2023, and in addition to AMR data, some countries started reporting AMU data. In the animal health sector, over 400,000 samples have been processed in Fleming Fund-supported sites, predominantly through passive surveillance and reflecting year-on-year increases (Figure 4). Antimicrobial resistance data contribution to the International Food and Agriculture Organization (FAO) Antimicrobial Resistance Monitoring System has been limited, as the first call for data only started in 2024 [39]. There were also 18,500 environmental health and 54,000 food safety samples processed. Burden-of-disease data have also been generated despite operational challenges in implementing SOPs and tools in programmatic LMIC settings, contributing to global modeling efforts and underscoring that clinically contextualized data systems add to laboratory surveillance alone.

**Figure 2.**
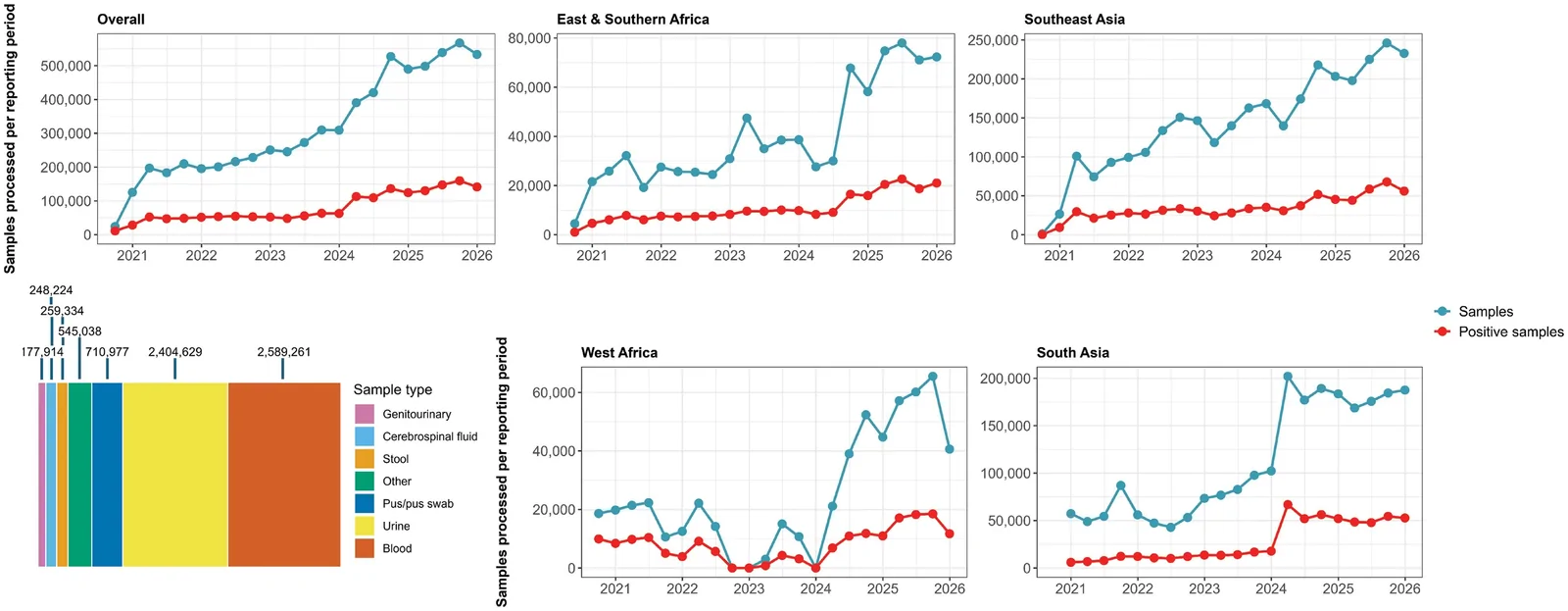
Human samples processed from Fleming Fund-supported sites. Shows data on the number of human samples processed overall by region (divided into East and Southern Africa, West Africa, South Asia, and Southeast Asia), with total numbers of samples processed per quarter. Blue lines indicate the total number of samples, and red lines indicate the number of positive samples. The treemap shows the proportion and number of samples overall by each sample type.

**Figure 3.**
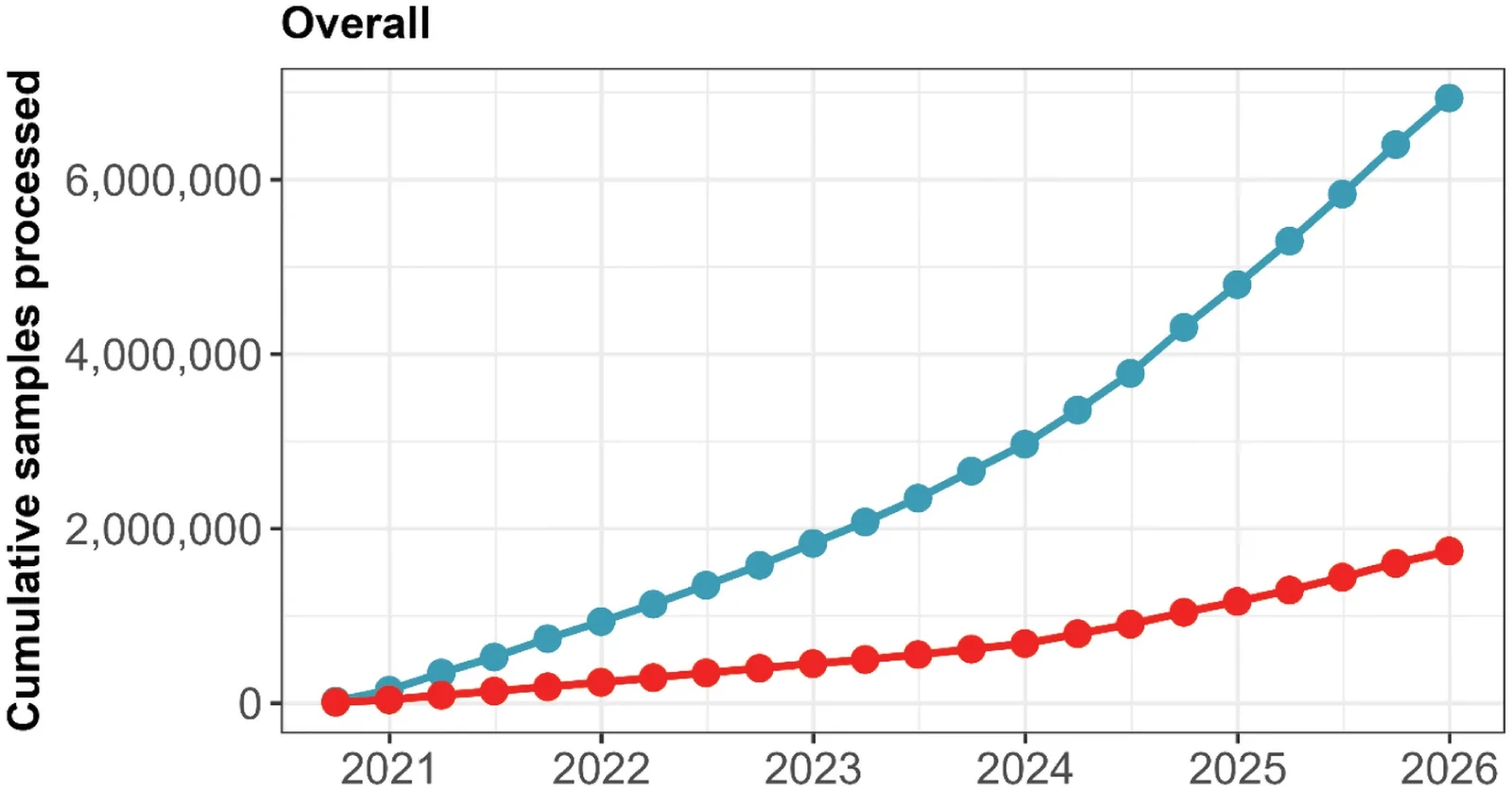
Cumulative human samples processed globally in Fleming Fund-supported sites. Blue lines indicate the total number of samples, and red lines indicate the number of positive samples.

**Figure 4.**
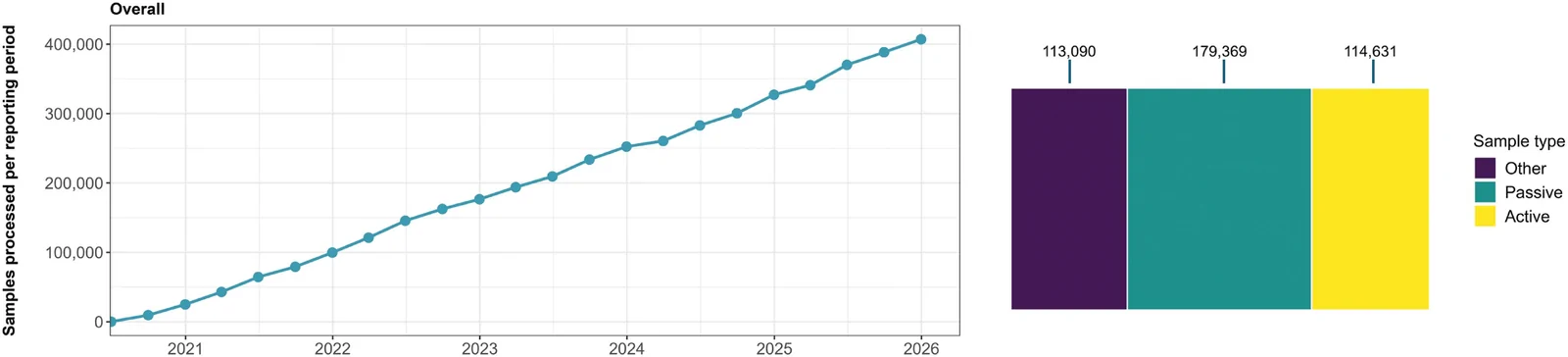
Animal samples processed from Fleming Fund-supported sites. Shows data on the number of animal samples processed in each quarter. The tree map shows the proportion of animal samples that were generated through passive sampling, active surveillance (ie sampling surveys), or other means.

World Health Organization’s GLASS does not directly evaluate laboratory performance; the quality and representativeness of AMR data remain dependent on laboratory quality assurance, as part of the laboratory quality management systems. Both Fleming Fund reporting processes and empirical evidence support an overall improvement in the quality of AMR data generated in Fleming Fund-supported sites and countries. For example, more national reference laboratories have achieved accreditation or are participating in EQA programs, more surveillance site-level laboratories have quality management systems and key quality-related SOPs available, and several rounds of EQA have occurred across all regions, with overall improvements in proficiency testing scores [15, 17, 40].

In addition to data reported through WHO's GLASS, increasing amounts of data describing AMR patterns and/or trends from Fleming Fund-supported countries are becoming available [41–45]. Studies suggest increasing AMR prevalence in some settings, although interpretation and generalization across regions remain challenging. For example, nationwide surveillance data from Bangladesh reported ∼12% prevalence of carbapenem-resistant Enterobacterales between 2017 and 2020 without a clear temporal decline [46]. Studies from Timor-Leste identified concerning levels of ESBL (Extended-Spectrum Beta-Lactamase) -producing and ciprofloxacin resistant Gram-negative isolates from children, and resistance to highest-priority critically important antimicrobials among poultry, illustrating variability across ecological and geographic contexts [47, 48]. More broadly, genomic and surveillance studies have documented diverse resistance mechanisms and expanding resistance determinants, but remain largely cross-sectional and heterogeneous in design, limiting inference on trends [25, 49].

Increases in reported AMR prevalence may partly reflect improved laboratory capacity, expanded sampling, and enhanced case ascertainment rather than true epidemiological change. The relative scarcity of published analyzes may reflect how resources were allocated to data analysis, synthesis, and incentives to publish data rather than an absence of data generation [50]. Consequently, current evidence remains insufficient to draw robust conclusions about AMR trends across Fleming Fund-supported countries, underscoring the need for sustained investment in analytical capacity alongside surveillance strengthening. In summary, the Fleming Fund contributed to increased and improved AMR surveillance data, in terms of both quantity and quality. However, this is variable and requires fundamental investment and resources to set up and maintain AMR data systems.

### How Has the Fleming Fund Strengthened Antimicrobial Resistance Surveillance Through Data Generation?

Strengthening AMR surveillance represents both a technically and an institutionally demanding undertaking [26, 51]. Fleming Fund-supported countries addressed a highly complex problem requiring laboratory infrastructure, skilled personnel, quality-assured microbiology, and data systems. At the same time, AMR surveillance is institutionally complex, spanning multiple disciplines, institutions, and sectors with differing mandates, incentives, and capacities [13]. At Fleming Fund inception, global guidance on operationalizing surveillance in LMICs was still evolving, meaning implementation relied on emerging frameworks, while evidence on sustainable surveillance models remained limited [11, 51]. Several assumptions made at the time the program was designed shaped its subsequent implementation, including that laboratory-centered surveillance was feasible across low-resource contexts accounting for local contexts, that strengthened capacity would lead to sustainability, and that increased data generation would translate into routine data use, although this would take time.

As expected, variability in health systems, contexts, and resources was a major challenge. Many countries began from a constrained baseline of laboratory infrastructure, where unreliable utilities, facility limitations, and laboratory quality management systems required foundational investment before surveillance could function effectively [52]. By contrast, some settings had laboratories providing AST for clinical samples, and AMR surveillance systems, but needed specific gap filling (eg establishing electronic laboratory data systems, automated bacteriological identification, and AST). The Fleming Fund was supporting countries to address basic infrastructure, such as unstable water and electricity, before testing could even occur while simultaneously procuring and installing automated bacteriology platforms, setting up LIMS, developing regional training centers, and supporting whole genome sequencing capacity (see Box 2 and Table 1). As expected, those countries where capacity was less took longer to start generating AMR data; for example, Senegal did not report AMR data until 2023, whereas countries with partly functional systems were able to achieve greater increases in AMR data reporting sooner.

Procurement delays, fragile supply chains, and consumable stock-outs impacting AMR testing, despite overall improvements in laboratory capabilities, were commonly cited as major challenges [28, 53, 54]. There were multiple reasons for this, including stocktaking, lack of local suppliers, long timelines for procurement, and importation delays. The Fleming Fund encouraged the use of local suppliers where possible to create a sustainable local market for reagents and consumables. However, reagents for automated systems proved difficult to source in some countries. In Phase 2, the Fleming Fund implemented a central procurement system for the reagents and consumables of automated analyzers to circumvent some of these challenges. The Fleming Fund approach of supporting automated blood culture was taken because it provides faster results with less effort for laboratory staff than manual blood culture methods. Automated identification and AST systems were supplied to reference laboratories to provide improvements in turnaround time, more precise identification and minimum inhibitory concentration values, and improve the degree of quality assurance [11, 55]. However, reagents for automated systems have proven difficult to source locally in some countries, and there have been concerns about maintenance, repair, and long-term affordability, threatening the sustainability of AMR surveillance.

In addition to laboratory and data infrastructure, human AMR surveillance data requires clinicians to sample patients. Many factors affected “demand generation,” including clinician trust in laboratory processes and results; turnaround times; antibiotic availability; user fees or availability of consumables, such as blood culture bottles; and knowledge and understanding of AMR [32]. This sometimes led to low demand and low sample throughput with consequences for sustainability. Success stories from the Fleming Fund include the use of AMS committees to encourage sampling and stimulate demand in Zambia and diagnostic stewardship interventions, including training for clinical staff, in Nepal and Laos that led to higher numbers and improved quality of samples [56].

Progress in data collation, analysis, and sharing often lags behind laboratory strengthening due to laboratory data infrastructure, fragmented reporting, and clinical metadata accompanying specimens, and improvements in diagnostic capacity do not automatically translate into usable data AMR data [26, 32]. The Fleming Fund experience from Nigeria illustrates this “data bottleneck,” where laboratories were producing AMR data that were not reported to national surveillance platforms until targeted investments addressed institutional barriers to data sharing, alongside training to strengthen data management skills. Similar challenges were overcome in Nepal, where limited interaction between clinicians, pharmacists, and laboratory personnel constrained use of microbiology results; implementation of a laboratory-clinic interface strengthening program helped create multidisciplinary platforms for data interpretation and improved reporting pathways. Together, these findings highlight a persistent “missing middle” commonly observed between data generation and data reporting in AMR surveillance systems, underscoring that such investments in data systems and analysis are essential complements to laboratory strengthening [50].

Ensuring consistent quality assurance for AMR data has remained challenging, reflecting both technical and systemic constraints. Laboratory quality improvement is often slow and fragile, influenced by factors beyond the reach of EQA programs, including reagent shortages, equipment and infrastructure maintenance, and broader health system constraints. In LMICs, requirements to strictly adhere to European Committee on Antimicrobial Susceptibility Testing or Clinical and Laboratory Standards Institute standards may be impractical, prompting locally adapted approaches, as seen in Timor-Leste's development of context-specific guidance. However, Fleming Fund EQA regional grants demonstrated measurable improvements in EQA performance across laboratories and settings and upskilled national reference laboratories to deliver their own EQA programs [15, 17, 40].

Human resources and financing are 2 challenges that apply across strengthening laboratory processes, demand generation, AMR data management, and quality assurance domains. Generating quality-assured AMR data depends on specialized skills that are often difficult to recruit and retain. Antimicrobial resistance surveillance literature repeatedly highlights workforce shortages, limited microbiology expertise, and the need for ongoing training and supervision to sustain consistent sampling, culture, and AST workflows [57]. The Fleming Fund experience mirrors this: High staff turnover and rotation resulted in training and quality systems gains “slipping back” as new staff entered posts faster than programs could re-train them, and in many settings, personnel covered multiple roles, sometimes with limited microbiology expertise. The Fleming Fund attempted to implement sustainable training models (eg train the trainer) where feasible and, in some settings (eg Nigeria), structured mentorship models to stabilize practice and improve quality management, illustrating that workforce strengthening must be continuous rather than episodic.

Health-system financing and broader resource constraints further shape AMR data generation, especially whether data can be produced at scale and sustained. Antimicrobial resistance data are dependent on predictable, recurring laboratory consumables (eg blood culture bottles, media, and antibiotic disks), laboratory equipment service contracts and maintenance, and staffing costs [58, 59]. These have often been covered by Fleming Fund grants in supported sites, with attempts to transition reagent procurement from full donor support to partial domestic funding (eg phased co-financing approaches in Timor-Leste). However, these were vulnerable to fiscal constraints, competing priorities, and shifting institutional buy-in, highlighting that sustained AMR data generation requires predictable budgets and governance arrangements that complement laboratory upgrades. Cost and affordability barriers are amplified in mixed public–private systems and where user fees can influence whether clinicians request microbiology tests and whether patients can access them; even modest per-test costs can suppress sampling volumes and bias surveillance toward better-resourced facilities [60]. The Fleming Fund experience underlines these requirements: In some settings, establishing basic infrastructure for AMR data production was itself a major achievement, for example average annual set-up costs per site were £112K in Zambia [61]. In many Fleming Fund-supported countries, sustained throughput depends on recurrent financing for consumables, maintenance, and staffing, and these averaged £90K annually per site in Zambia [58, 61].

Challenges to AMR data production in the animal health sector reflect both shared and distinct constraints compared with human health. Many of the systemic barriers identified in human AMR systems, (eg laboratory infrastructure, workforce, fragmented data systems, and procurement bottlenecks) are also present in animal health and are often intensified by comparatively lower funding [62, 63]. However, in settings with commercial livestock production, microbiological testing may be privately financed as part of routine business practice, generating substantial volumes of susceptibility data. The primary challenge in such settings is integration of commercially produced results into national surveillance platforms, including harmonization of laboratory methods and data systems (eg WHONET). Conversely, in predominantly smallholder or subsistence systems, awareness, testing demand, and financial incentives may be insufficient to sustain routine microbiology, limiting data production altogether. Additional constraints include regulatory barriers to isolate transfer, limited in-country molecular capacity, and budgetary gaps due to competing priorities in the animal health sector [64]. Collectively, these experiences underscore that animal health AMR surveillance requires context-specific strategies to ensure that data generated, whether publicly or privately financed, are standardized and sustainably incorporated into national One Health surveillance systems [13, 65, 66].

### What Are the Remaining Gaps and Future Strategies for Antimicrobial Resistance Data Generation?

Despite substantial and measurable progress in laboratory strengthening and AMR data production through the Fleming Fund and other mechanisms, AMR surveillance systems in LMICs are fragile and unevenly institutionalized. The Fleming Fund has demonstrated that building bacteriology capacity and supporting international reporting is feasible across diverse settings, but required significant time, resource, and multistranded interventions; sustainability without ongoing donor support remains uncertain. Without integration into national financing and universal health coverage packages, diagnostic and surveillance gains are unlikely to be sustained. In many high-income countries, AMR surveillance has emerged as a byproduct of established diagnostic clinical microbiology services. The cost of maintaining a standard national AMR surveillance system is estimated to exceed 1%–5% of the total domestic government health expenditure in many LMICs [67, 68]. By ensuring microbiological testing first and foremost guides patient care, and facilities can use their own data, we can improve patient outcomes and continue AMR surveillance [69].

A second continuing challenge lies in data representativeness and access. Surveillance networks in many countries remain concentrated in tertiary or urban referral hospitals, generating isolate-based data that are incompletely linked to population denominators, or clinical syndromes and outcomes, limiting the geographic and socioeconomic representativeness of surveillance [14]. This imbalance reflects a broader constraint in access to diagnostic services: Nearly half of the global population lacks access to even basic diagnostics [70]. Without expanded access to microbiology at district and primary care levels, AMR data may continue to be skewed toward more severe or hospital-managed infections. The surveillance challenge is therefore not only to produce more data but also to generate data that are epidemiologically meaningful, population based, and linked to antimicrobial use and clinical outcomes. Modeling exercises, such as GRAM, underscore how incomplete LMIC datasets and passive isolate-based surveillance constrain burden estimation and forecasting, reinforcing the need for stronger epidemiological integration.

Digital maturity and data integration represent another critical frontier. Fleming Fund investment in training, LIMS, and analysis tools has improved standardization and skills, but more interoperability (such as with clinical records and antimicrobial consumption data) will be beneficial. There are calls for diagnostic ecosystems that are digitally enabled and aligned with FAIR (Findable, Accessible, Interoperable, and Reusable) data principles, yet many AMR datasets remain siloed, incomplete, or poorly harmonized. Artificial intelligence and advanced analytics offer promise for real-time resistance prediction, clinical decision support, and surveillance optimization, but their performance depends fundamentally on high-quality, linked, and representative data [71]. In this respect, AMR surveillance faces a paradox: Technological capability is advancing rapidly, while access to foundational bacteriology and data systems remains uneven. Similarly, whole-genome sequencing and regional genomic hubs have expanded rapidly in some LMICs, catalyzed by COVID-19 investments and Fleming Fund grants, but integration of genomic data into routine surveillance and national reporting remains nascent even in high-income settings [72, 73]. There is potential for innovative microfluidic, molecular, sequencing, and decentralized point-of-care diagnostics, as well as cloud-based analytics; however without robust frameworks for data collection, quality management, and sustainable financing, such technologies risk widening inequities [74]. Antimicrobial resistance surveillance must also innovate beyond vertical approaches to be embedded within broader diagnostic platforms, enabling integration with routine health-system workflows and universal health coverage strategies, and supporting efficiency and sustainability; the tail of surveillance should not wag the dog of patient care [75].

Alternative strategies for AMR surveillance may be more sustainable or complement routine laboratory-based reporting; however, the purpose of surveillance must always be clear. Research-based sentinel surveillance models, often embedded within academic or reference laboratory networks, can generate high-quality, standardized datasets with linked clinical data, supporting more accurate burden and resistance mechanism analysis [23, 25]. Active surveillance approaches, including prospective case finding, structured sampling protocols, and periodic or targeted population surveys, can reduce biases, improve representativeness, and be more efficient [76, 77]. Event-based surveillance, genomic cluster detection, and integrated antimicrobial consumption monitoring offer additional lenses through which resistance trends can be interpreted [41]. Hybrid models that combine routine passive reporting with periodic active or research-driven surveillance may provide a pragmatic pathway in LMICs, balancing feasibility with epidemiological robustness while enabling more meaningful linkage between resistance data, clinical outcomes, and antimicrobial use.

Finally, institutional and political prioritization challenges remain in many settings. Laboratory capacity strengthening has been accompanied by human resources, professional networks, governance, and shifts in norms. However, diagnostics and surveillance historically receive a small proportion of research and development funding, despite being foundational to stewardship, infection prevention, and antimicrobial development. Forward-looking strategies should consider moving beyond laboratory strengthening toward integrated, digitally enabled, and embedded surveillance systems that can also impact clinical care. This includes expanding primary-level diagnostic access, harmonizing standards across sectors, investing in genomic and digital capacity alongside quality systems, and embedding surveillance within domestic financing frameworks. Such systemic consolidation could help maintain technically impressive gains achieved over the past decade.

## CONCLUSIONS

Laboratory surveillance plays a foundational public health function at local, national, and global levels, underpinning clinical care, stewardship, outbreak detection and health security, and, ultimately, AMR prevention. Sustained investment is essential to ensure that surveillance systems remain functional, representative, and responsive to evolving AMR threats. The Fleming Fund has demonstrated that coordinated, multicountry, and multisectoral investment, linking laboratory capacity, workforce development, data systems, and governance, can catalyze meaningful progress and offers a practical model for collective global action. Technical gains will only translate into lasting impact when momentum is maintained, recurrent financing is secured, and continuing challenges (eg representativeness, digital integration, and One Health coordination) are addressed. Surveillance data also need to be consistently and critically analyzed and used to inform clinical guidelines, antimicrobial policies, and resource allocation. The next phase of the global AMR response must prioritize surveillance systems to ensure sustainable generation, sharing, and effective use of high-quality data.
